# Cross-national risk factors for childbirth-related PTSD: Findings from the INTERSECT study

**DOI:** 10.1017/S0033291725102298

**Published:** 2025-11-17

**Authors:** Jonathan E. Handelzalts, Susan Ayers, Rebecca Webb, Georgina Constantinou, Grace Lucas, Christopher Grollman, Shay Ohayon, Natalia Awad Sirhan, Kathleen Baird, Márcia Baldisserotto, Ramish Batool, Shahida Batool, Rafael A. Caparros-Gonzalez, Genesis Chorwe-Sungani, Andri Christoforou, Soledad Coo, Raquel Costa, Pelin Dikmen-Yildiz, Barbora Ďuríčeková, Bohdana Dušová, Violeta Enea, Susan Garthus-Niegel, Hanna Grundström, Oye Gureje, Eleni Hadjigeorgiou, Silje Marie Haga, Antje Horsch, Chiara Ionio, Gabija Jarašiūnaitė-Fedosejeva, Julie Jomeen, Maria Kazmierczak, Joan Lalor, Maja Milosavljevic, Ursula Nagle, Sandra Nakić Radoš, Katri Nieminen, Bibilola Damilola Oladeji, Flavia Osório, Paulina Pawlicka, Yoav Peled, Tiago Miguel Pinto, Valentine Rattaz, Olga Riklikienė, Julia Schellong, Valgerður Lísa Sigurðardóttir, Narenda Singh Thagunna, Mariza Theme Filha, Zuzana Škodová, Petra Stebelová, Tjasa Stepisnik Perdih, Robert Stewart, Emma Marie Swift, Kristiina Uriko, Zahir Vally, Milica Vezmar, Haya S. Zedan, Maja Žutić, the INTERSECT Consortium

**Affiliations:** 1 School of Behavioral Sciences, Academic College of Tel, Aviv-Yafo, Israel; 2 Centre for Maternal and Child Health Research, City St George’s, University of London, London, UK; 3 Department of Psychology, Bar-Ilan University, Ramat-Gan, Israel; 4 Psychology Department, Universidad del Desarrollo, Santiago, Chile; 5 School of Nursing and Midwifery, Faculty of Health, University of Technology, Sydney, Australia; 6 Universidade Federal do Rio de Janeiro, Instituto de Psicologia, Departamento de Psicometria, Rio de Janeiro, Brazil; 7 Beaconhouse School System, Beaconhouse Boys Campus, Lahore, Pakistan; 8 Department of Psychology, GC University, Lahore, Pakistan; 9 Faculty of Health Sciences, Department of Nursing, University of Granada, and Instituto de Investigación Biosanitaria ibs.GRANADA, Granada, Spain; 10 Department of Mental Health Nursing, School of Nursing, Kamuzu University of Health Sciences, Blantyre, Malawi; 11 Department of Social and Behavioral Sciences, European University Cyprus, Nicosia, Cyprus; 12 Lusófona University, HEI-Lab: Digital Human-Environment Interaction Labs, Lisboa, Portugal; 13 Department of Psychology, Kirklareli University, Kirklareli, Türkiye; 14 Department of Midwifery, Jessenius Faculty of Medicine in Martin, Comenius University, Bratislava, Slovakia; 15 Faculty of Medicine, University of Ostrava, Ostrava, Czech Republic; 16 Department of Psychology, Alexandru Ioan Cuza University, Iasi, Romania; 17 Department of Childhood and Families, Norwegian Institute of Public Health, Oslo, Norway; 18 Institute and Policlinic of Occupational and Social Medicine, Faculty of Medicine, TUD Dresden University of Technology, Dresden, Germany; 19 Department of Obstetrics and Gynecology in Norrköping, Department of Biomedical and Clinical Sciences, Linköping University, Linköping, Sweden; 20 Department of Psychiatry, College of Medicine, University of Ibadan, Ibadan, Nigeria; 21 Department of Nursing, School of Health Sciences, Cyprus University of Technology, Limassol, Cyprus; 22 Regional Centre for Child and Adolescent Mental Health, Eastern and Southern Norway, Oslo, Norway; 23 Institute of Higher Education and Research in Healthcare Sciences, University of Lausanne, Lausanne, Switzerland; and Department Woman-mother-child, Lausanne University Hospital, Lausanne, Switzerland; 24 Trauma Research Unit, Department of Psychology, Catholic University of Milan, Milan, Italy; 25 Department of Psychology, Faculty of Social Sciences, Vytautas Magnus University, Kaunas, Lithuania; 26 Faculty of Health, Southern Cross University, Gold Coast, Australia; 27 Faculty of Social Sciences, Institute of Psychology, University of Gdansk, Gdansk, Poland; 28 School of Nursing and Midwifery, Trinity College, Dublin, Ireland; 29 Institute of Mental Health, Belgrade, and Faculty of Medicine, University of Belgrade, Belgrade, Serbia; 30 Department of Psychology, Catholic University of Croatia, Zagreb, Croatia; 31 Department of Neurosciences and Behavioral Sciences, Ribeirão Preto Medical School, University of Sao Paulo, Brazil; 32 The Helen Schneider Hospital for Women, Rabin Medical Center, Faculty of Medicine, Tel-Aviv University, Tel-Aviv, Israel; 33 Faculty of Nursing, Department of Nursing, Lithuanian University of Health Sciences, Kaunas, Lithuania; 34 Department of Psychotherapy and Psychosomatic Medicine, Faculty of Medicine, Technische Universität Dresden, Dresden, Germany; 35 Faculty of Nursing and Midwifery, University of Iceland, Reykjavík, National University Hospital, Reykjavík, Iceland; 36 Psychdesk Foundation, Kathmandu, Nepal; 37 Department of Epidemiology and Quantitative Methods on Health, National School of Public Health, Oswaldo Cruz Foundation, Rio de Janeiro, Brazil; 38 School of Advanced Social Studies[CMT5], Nova Gorica, Slovenia; 39 Division of Psychiatry, University of Edinburgh, Edinburgh, UK; 40 School of Natural Sciences and Health, Department of Psychology and Behavioral Sciences, Tallinn University, Tallinn, Estonia; 41 Department of Clinical Psychology, United Arab Emirates University, Abu Dhabi, United Arab Emirates; 42 Department of Public Health, College of Health Sciences, Saudi Electronic University, Riyadh, Saudi Arabia

**Keywords:** Childbirth, Postpartum, PTSD, Risk Factors

## Abstract

**Background:**

Childbirth-related post-traumatic stress disorder (CB-PTSD) is an underrecognized condition with consequences for mothers and infants. This study aimed to determine risk factors for CB-PTSD symptoms across countries within a stress–diathesis framework, focusing on antenatal, birth-related, and postpartum predictors.

**Methods:**

The INTERSECT cross-sectional survey (April 2021–January 2024) included 11,302 women at 6–12 weeks postpartum. The study was carried out across maternity services in 31 countries. Outcomes were CB-PTSD diagnosis, symptom severity, and perceived traumatic birth, assessed with the City Birth Trauma Scale. Multiple risk factors were assessed, including preexisting vulnerability, pregnancy, birth, and infant-related factors. All models were adjusted for country-level variation as a random effect.

**Results:**

Models explained substantial variance across all outcomes (conditional *R*^2^ = 0.53–0.58). Negative birth experience was the strongest predictor (e.g. odds ratio [OR] = 0.82, 95% confidence interval [CI] = 0.80–0.84 for diagnosis). Ongoing maternal complications predicted both CB-PTSD diagnosis and symptoms (e.g. OR = 1.61, 95% CI = 1.41–1.84), and major infant complications were associated with CB-PTSD diagnosis (OR = 1.63, 95% CI = 1.29–2.07). Reports of perceived danger to self or infant (criterion A) were linked to higher CB-PTSD symptoms and traumatic birth ratings (e.g., *β* =0.25, 95% CI = 0.21–0.29). Other predictors reached significance but showed small effects.

**Conclusions:**

Findings support a stress–diathesis framework, showing that while pre-existing vulnerabilities contribute, birth-related stressors exert the strongest influence. Trauma-informed maternity care should prioritize these factors, with attention to women’s appraisals of birth.

## Introduction

Despite the culturally positive perception of childbirth (Horesh, Garthus-Niegel, & Horsch, 2021), up to one-third of mothers report their experiences as traumatic (Ayers, Harris, Sawyer, Parfitt, & Ford, 2009; Ayers, Handelzalts, Webb, et al., under review; O’Donovan et al., 2014; Stramrood, et al., 2011). Some go on to develop childbirth-related post-traumatic stress disorder (CB-PTSD), with a recent meta-analysis estimating its prevalence at 4.7% (Heyne et al., 2022). CB-PTSD has been associated with various adverse outcomes for mothers, infants, and family well-being and health (Horsch et al., 2024).

The public health significance of CB-PTSD is underscored by the number of births each year. In 2023 alone, ~140 million children were born worldwide (UN, 2024). Based on the estimated prevalence, this means that around 6.5 million women may develop CB-PTSD every year, underscoring its profound implications for public health and emphasizing the need for greater awareness and support.

According to the diathesis-stress model of CB-PTSD (Ayers, Bond, Bertullies, & Wijma, 2016), risk factors can be categorized into: (1) **antenatal factors**, such as maternal characteristics and mental health, trauma history, and predisposing demographic factors; (2) **birth factors**, including subjective birth experiences, operative births, and obstetric complications; and (3) **postpartum factors**, such as comorbid mental health conditions (e.g. depression and anxiety) and ongoing mother and infant physical complications.

Systematic reviews and meta-analyses (Ayers et al., 2016; Andersen et al., 2012; Dekel, Stuebe, & Dishy, 2017; El Founti Khsim Martínez Rodríguez, Riquelme Gallego, Caparros-Gonzalez, & Amezcua-Prieto, 2022; Grekin & O’Hara, 2014; Heyne et al., 2022; Kranenburg et al., 2023) consistently identify the **birth factor** of negative subjective birth experiences as the strongest risk factor, regardless of the level of obstetric complications (Andersen et al., 2012; Dekel et al., 2017). Additionally, obstetric interventions such as emergency cesarean sections and instrumental vaginal births increase risk (Andersen et al., 2012; El Founti Khsim et al., 2022; Heyne et al., 2022; Orovou, Antoniou, Zervas, & Sarantaki, 2025). **Antenatal factors** are also crucial contributors, including prenatal depression, fear of childbirth, trauma history, and preexisting mental health conditions (Ayers et al., 2016; El Founti Khsim et al., 2022). **Postpartum risk factors** include inadequate support, postpartum depression, and maladaptive coping (Ayers et al., 2016).

Despite extensive research on CB-PTSD, mostly in high-income countries, cultural and regional factors may limit the generalizability of identified risk factors. The diathesis-stress model emphasizes the cumulative influence of multiple factors across the perinatal period, rather than a single vulnerability. The aim of this study is to examine risk factors within a unified model based on the stress-diathesis model (Ayers et al., 2016) in a large, diverse international sample. We assessed three trauma-related outcomes: possible CB-PTSD diagnosis, symptom severity, and perceived traumatic birth.

## Methods

### Design

INTERSECT is a cross-sectional survey examining traumatic birth and CB-PTSD in women 6–12 weeks postpartum (mean = 8.5, standard deviation [SD] = 1.9 weeks). The study protocol was preregistered (Ayers et al., 2021). Data for INTERSECT (Ayers, Handelzalts, & INTERSECT Consortium, 2025) were collected between April 2021 and January 2024, and involved 11,302 participants from 31 countries (see eTable 1 in the Supplementary Materials for sample size and selected demographic data by country). The dataset is available on request via the UK Data Service (Ayers, Handelzalts, & the INTERSECT Consortium, 2021–2024).

### Participants

Inclusion criteria required participants to have given birth within the past 6–12 weeks and be legal adults in their country of residence (16+ or 18+ years). The sample comprised 10,086–10,130 women (the number of participants varied, depending on analysis – see flow chart eFigure 1 in the Supplementary Materials) from 30 countries. As shown in Table 1, most participants were aged 30–34 years (33.72%), held higher education qualifications (56.84%), reported an average income (relatively to the country; 56.85%), were married (68.58%), and belonged to the majority ethnic group in their country (83.44%).

**Table 1.** Sociodemographic characteristics of the sample (*N* = 10,130)

Variable	*N* (%)
Age (years)	
<20	194 (1.92%)
20–24	1,086 (10.72%)
25–29	2,822 (27.86%)
30–34	3,416 (33.72%)
35–39	1,998 (19.72%)
40+	614 (6.06%)
Education	
No formal education	144 (1.42%)
Elementary	820 (8.10%)
Secondary	3,408 (33.64%)
Higher education	5,758 (56.84%)
Income	
Below average	1,907 (18.83%)
Average	5,759 (56.85%)
Above average	2,464 (24.32%)
Relationship status	
Married	6,947 (68.58%)
Living with a partner	2,651 (26.17%)
In relationship	133 (1.31%)
Single	294 (2.90%)
Widowed	13 (0.13%)
Separated/divorce	78 (0.77%)
Other	14 (0.14%)
Ethnicity	
Majority	6,771 (83.44%)
Minority	817 (10.07%)
Not sure	527 (6.49%)

### Procedures

Ethical approval was obtained by the principal investigators in each country. Inclusion criteria, sampling procedures, and survey questions were consistent across countries (Ayers et al., 2021).

Participants were recruited in pregnancy or postpartum through routine maternity care services – for example, hospitals, clinics, and birth centers. To minimize self-selection bias and improve representativeness, recruitment through social media was avoided, except in Slovenia and Norway, where it was used alongside the standard protocol; however, the recruitment mode was not documented separately in these countries. Research teams contacted participants in person, by phone, video call, or email, and provided study information. Those who agreed to participate provided informed consent. Surveys were completed using online forms, paper questionnaires, or telephone interviews.

The INTERSECT survey was originally developed in English. Validated translations were used where available, or survey measures were translated and adapted according to standard cultural adaptation procedures (Wild et al., 2005).

## Measures

### Outcome variables

CB-PTSD was measured using the City Birth Trauma Scale (Ayers, Wright, & Thornton, 2018), which uses the Diagnostic and Statistical Manual of Mental Disorders, Fifth Edition (DSM-5) criteria to assess PTSD symptoms due to labor, birth, or immediate postpartum events. Symptom frequency in the past week is rated from 0 (‘not at all’) to 3 (‘5 or more times’). The scale also assesses distress, impairment, and duration of symptoms. CB-PTSD cases are calculated according to the DSM-5 PTSD diagnostic criteria. The scale has good reliability and psychometric validity across translations (Nakić Radoš, Matijaš, Kuhar, Anđelinović, & Ayers, 2020; Osorio, Darwin, Bombonett, & Ayers, 2022; Sandoz et al., 2022). Internal consistency in this sample was high (McDonald’s *ω* = 0.94). Two scores from this scale were used as dependent variables: **the total symptom score** (range 0–60) and a binary **CB-PTSD probable diagnosis** (yes/no).

Perceived traumatic birth was assessed using a single item on the extent to which participants experienced their **birth as traumatic** (Overall, how traumatic did you find your birth) on an 11-point scale, ranging from 0 (‘*not at all*’) to 10 (‘*extremely*’).

### Risk factors

Birth experience was assessed using the Birth Satisfaction Scale-Revised (Martin & Martin, 2014), a 10-item scale encompassing quality of care, personal attributes, and stress during labor. Responses are rated on a Likert scale ranging from 0 (‘*strongly disagree*’) to 4 (‘*strongly agree*’) with total scores ranging from 0 to 40. Internal consistency in this sample was high (McDonald’s *ω* = 0.87).

Previous trauma was assessed dichotomously (yes/no) using the trauma checklist from the Post-Traumatic Stress Diagnostic Scale (Foa, Cashman, Jaycox, & Perry, 1997), which assesses prior traumas – for example, life-threatening illness, physical assault, sexual assault, military combat or experience in a war zone, child abuse, accident, natural disaster, and other trauma. Endorsing any trauma was scored as yes. Additional items assessed previous traumatic birth and pregnancy loss or stillbirth (yes/no).

History of psychological problems and treatment was assessed using items evaluating both current and/or past mental health diagnoses (yes/no), treatment (yes/no), and the type of treatment received (medication, professional support, or both).

Demographic and obstetric information encompassed age, ethnicity, household income, education, relationship status, and immigration status. Obstetric data included the number of children (before this birth), gestational age, birth mode (vaginal/assisted vaginal birth/emergency cesarean/elective cesarean), number of birth companions, as well as perceived level of support from them (measured on a 0–4 Likert scale), and maternal or infant complications (major/minor/none). Obstetric information was self-reported in all countries except Germany, where data were obtained from medical records.

### Data handling and governance

Data were collected and coded consistently across countries using a standardized data dictionary. Anonymized data were uploaded to City St. George’s, University of London’s data server. A data protection impact assessment was conducted and approved, and research governance and data-sharing agreements were established between host and partner institutions.

### Data analysis

All analyses were conducted in R (Core Team, 2021) using the ‘lme4’ package (Bates et al., 2015). Part *R*^2^ were calculated using ‘partR2’ package for R (Stoffel, Nakagawa, & Schielzeth, 2021). Data from each country were harmonized and linked as described elsewhere (Ayers et al., Under review). Participants from Switzerland were excluded from the analysis due to missing data on the number of birth companions. Missing values were not at random and, therefore, not imputed, and participants with missing values were excluded (see flowchart eFigure 1 in the Supplementary Materials). All continuous independent variables were centered. Categorical variables were re-coded using effect coding.

Separate analyses were conducted for dependent variables of CB-PTSD diagnosis, CB-PTSD total symptoms, and traumatic birth. Due to missing values, there are different numbers of participants between models. To construct the final models, a process of variable selection was conducted. We conducted four separate analyses for each dependent variable, systematically examining the significant independent variables within each category as follows (see Supplementary Materials):

1. Background variables, that is, age, level of education, level of income, number of children, immigration status, resident area, relationship status, previous trauma, previous or current mental health diagnosis, current or previous mental health treatment, and type of mental health treatment (Supplementary eTable 2).
2. Pregnancy variables, that is, previous pregnancy loss and previous birth trauma (Supplementary eTable 3).
3. Birth variables, that is, birth method, maternal complications during birth, ongoing maternal complications, DSM-5 criterion A (believed that she or the baby would be severely injured or die during birth), number of birth companions, perceived level of support from birth companions, and birth experience (Supplementary eTable 4).
4. Infant-related variables, that is, infant complications during birth, ongoing infant complications, and gestation (Supplementary eTable 5).

Next, all significant variables from these models were entered together into the final three models, one model for each dependent variable.

To account for the variability of the effects between countries, we compared three covariance matrix structures for each model: (1) a random intercept model; (2) a random intercept + slopes model with a diagonal covariance structure; and (3) a random intercept + slopes model with an unstructured covariance structure. Models were selected based on convergence, absence of singularity, and superior fit. SDs of the random slopes for each model are reported in Supplementary eTable 6.

Based on the results of the first stage of analyses (background variables), the number of children was retained and included as a predictor in the final models. We repeated the analyses with a binary variable representing parity (as reported in many studies). As two models demonstrated a slightly better fit when using the number of children, this specification was retained. Importantly, the overall pattern of results remained unchanged when parity was used as a predictor.

## Results

First, we explored the bivariate correlations between dependent variables. Results revealed significant positive correlations between CB-PTSD diagnosis and CB-PTSD symptoms (*r* = 0.54, *p* < .001), CB-PTSD diagnosis and perceived traumatic birth (*r* = 0.31, *p* < .001), and CB-PTSD symptoms and perceived traumatic birth (*r* = 0.47, *p* < .001).

### Risk factors for a probable diagnosis of CB-PTSD

The random slopes of the final model predicting CB-PTSD diagnosis were defined for number of children, previous trauma, current mental health diagnosis, level of support from birth companion, ongoing mother and infant complications, birth experience (BSS-R score), and major infant complications during birth (see Equation 1 in the Supplementary Materials), with diagonal covariance structure (Akaike Information Criterion [AIC] = 3009.8, Bayesian Information Criterion [BIC] = 3168.7).

The fixed effects of the final model are shown in Table 2. Significant risk factors for CB-PTSD diagnosis were previous birth trauma and other traumas, current mental health diagnosis, ongoing maternal complications, current infant complications, number of birth companions, and major infant complications during the birth. Negative birth experience was associated with CB-PTSD diagnosis.

**Table 2.** Predictors of CB-PTSD diagnosis (fixed effects, *N* = 10,116)

	OR	95% CI	*z*	*P*	Part *R*^2^
Number of children	0.93	0.81, 1.06	−1.13	.257	0.00
Any previous trauma	1.36	1.14, 1.62	3.43	<.001	0.01
Previous mental health diagnosis	1.12	0.94, 1.34	1.24	.215	0.00
Current mental health diagnosis	1.36	1.09, 1.68	2.79	.005	0.00
Previously received mental health treatment	1.09	0.91, 1.32	0.94	.349	0.00
Previous traumatic birth	1.24	1.07, 1.42	2.95	.003	0.00
Ongoing maternal complications	1.61	1.41, 1.84	7.00	<.001	0.01
Number of birth companions	1.41	1.10, 1.79	2.75	.006	0.00
Level of support from birth companion	0.89	0.77, 1.03	−1.61	.106	0.00
Birth experience	0.82	0.80, 0.84	−15.70	<.001	0.11
Major infant complications during birth	1.63	1.29, 2.07	4.11	<.001	0.01
Ongoing infant complications	1.35	1.08, 1.69	2.64	.008	0.00

The conditional *R*^2^ of the final model was 0.58, and the marginal *R*^2^ was 0.36, suggesting that both fixed and random effects accounted for a substantial proportion of variance in PTSD diagnosis. The model had a significantly better fit than a model with only a random intercept (AIC = 3071.5, BIC = 3172.6, *χ*^2^(8) =77.65, *p* < .001). This indicates significant variation in slopes across countries in the random effect variables. Additionally, the model fit of the final model was not significantly different from a random intercept and slopes model with an unstructured covariance matrix (AIC = 3127.1, BIC = 3784.3, *χ*^2^(69) =20.71, *p* = 1.000). This means that the more complex model, which addressed the correlations between slopes, did not provide a better fit to the data than the simpler model, suggesting that the added complexity was unnecessary.

### Risk factors for CB-PTSD symptom severity

The final model predicting CB-PTSD symptoms is detailed in Equation 2 in the Supplementary Materials. Random slopes were defined for all predictors, except for relationship status (partner), current and past mental health treatment, birth method (elective cesarean), and major infant complications during the birth, with a diagonal covariance structure (AIC = 69867.0, BIC = 70098.0).

The fixed effects are shown in Table 3. Significant risk factors for CB-PTSD symptoms were any previous trauma, current mental health diagnosis, currently or previously receiving mental health treatment, previous traumatic birth, ongoing maternal complications, believing she or her baby would be injured or die during birth, number of birth companions, major infant complications during birth, and ongoing infant complications. Protective factors were the number of children, level of support from birth companions, and positive birth experience.

**Table 3.** Predictors of CB-PTSD symptoms (fixed effects, *N* = 10,130)

	*β*	95% CI	*T*	*P*	Part *R*^2^
Number of children	−0.08	−0.12, −0.04	−3.72	<.001	0.00
Relationship status – partner	−0.00	−0.04, 0.02	−0.31	.757	0.00
Any previous trauma	0.12	0.08, 0.16	6.12	<.001	0.01
Current mental health diagnosis	0.25	0.20, 0.29	10.05	<.001	0.04
Currently receiving mental health treatment	0.08	0.02, 0.13	2.83	.005	0.00
Previously received mental health treatment	0.08	0.03, 0.12	3.27	.001	0.00
Previous traumatic birth	0.10	0.05, 0.15	4.11	<.001	0.01
Birth method - elective cesarean	−0.03	−0.05, 0.00	−1.76	.078	0.00
Major maternal complications during birth	0.04	−0.01, 0.08	1.45	.147	0.00
Ongoing maternal complications	0.19	0.13, 0.25	6.32	<.001	0.04
During birth – believed she or the baby injured	0.20	0.13, 0.26	5.91	<.001	0.05
During birth – believed she or the baby would die	0.23	0.15, 0.32	5.36	<.001	0.06
Number of birth companions	0.12	0.05, 0.19	3.50	<.001	0.00
Level of support from birth companion	−0.11	−0.19, −0.04	−2.87	.004	0.01
Birth experience	−0.58	−0.68, −0.48	−11.56	<.001	0.16
Major infant complications during birth	0.04	0.00, 0.07	2.19	.029	0.00
Ongoing Infant complications	0.05	0.00, 0.11	2.23	.026	0.00

The conditional *R*^2^ of the final model was 0.55, and the marginal *R*^2^ was 0.27, suggesting that both fixed and random effects accounted for a substantial proportion of variance in PTSD symptom severity. A comparison of the final model with a model incorporating additional random slopes revealed no significant improvement in fit (AIC = 69,866, BIC = 70,118, *χ*^2^(3) =7.18, *p* = .066), thereby supporting the selection of the more parsimonious model. In addition, the final model had a significantly better fit than a model with fixed effects and a random intercept only (AIC = 71,002, BIC = 71,146, *χ*^2^(12) =1158.8, *p* < .001), suggesting there is variation in slopes between countries in the random effect variables. An alternative model specification using an unstructured covariance structure did not converge and was, therefore, not further analyzed.

### Risk factors for perceived traumatic birth

The final model predicting perceived traumatic birth is detailed in Equation 3 in the Supplementary Materials. In this model, random slopes were defined for all predictors, except for number of children and birth method – assisted vaginal, ongoing maternal complications, major infant complications during birth, and ongoing infant complications, with diagonal covariance structure (AIC = 44033.9, BIC = 44207.2).

The fixed effects are shown in Table 4. Significant risk factors for perceived traumatic birth were assisted vaginal birth, emergency cesarean, major maternal complications during the birth, ongoing maternal complications, believing she or her baby would be injured or die during birth (criterion A), and major infant complications during birth. Protective factors were the number of children, elective cesarean birth, and positive birth experience.

**Table 4.** Predictors of perceived traumatic birth (fixed effects, *N* = 10,086)

	*β*	95% CI	*t*	*P*	Part *R*^2^
Number of children	−0.09	−0.12, −0.06	−6.54	<.001	0.00
Any previous trauma	0.03	−0.01, 0.07	1.37	.169	0.00
Currently mental health diagnosis	0.00	−0.04, 0.04	0.05	.962	0.00
Birth mode					
Vaginal (ref.)	-	-	-	-	-
Assisted vaginal	0.11	0.06, 0.16	4.06	<.001	0.00
Emergency cesarean	0.17	0.10, 0.25	4.54	<.001	0.02
Elective cesarean	−0.26	−0.33, −0.19	−7.67	<.001	0.00
Major maternal complications during the birth	0.12	0.07, 0.16	5.35	<.001	0.02
Ongoing maternal complications	0.04	0.01, 0.07	2.49	.013	0.00
During birth – believed she or the baby would be injured	0.17	0.10, 0.24	4.74	<.001	0.05
During birth – believed she or the baby would die	0.19	0.14, 0.25	6.70	<.001	0.05
Birth experience	−0.87	−0.98, −0.75	−15.12	<.001	0.31
Major infant complications during the birth	0.05	0.02, 0.09	3.37	<.001	0.00
Ongoing infant complications	0.03	0.00, 0.06	1.68	.093	0.00

The conditional *R*^2^ of the final model was 0.53, and the marginal *R*^2^ was 0.37, suggesting that both fixed and random effects accounted for a substantial proportion of variance in perceived birth trauma. Comparing the final model with a model incorporating additional random slopes revealed no significant improvement in fit (AIC = 44,039, BIC = 44,207, *χ*^2^(4) =3.28, *p* = .513); consequently, the more parsimonious model was retained. In addition, the final model demonstrated a significantly better fit than a model with fixed effects and a random intercept only (AIC = 44,561, BIC = 44,677, *χ*^2^(8) =543.53, *p* < .001), indicating that slopes vary significantly between countries for the defined predictors. An alternative model specification using an unstructured covariance structure resulted in singularity and was not further analyzed.

## Discussion

Although CB-PTSD has been widely studied (Andersen et al., 2012; Ayers et al., 2016; El Founti Khsim et al., 2022; Gerkin & O’Hara, 2014; Heyne et al., 2022; Kranenburg et al., 2023), this is the first study to examine risk factors in an international sample across multiple countries with standardized measures using the diathesis-stress model that emphasizes the cumulative influence of multiple factors across the perinatal period, rather than a single vulnerability. A key strength is the examination of three outcome variables: CB-PTSD diagnosis, CB-PTSD symptoms, and perceived traumatic birth. This multidimensional approach enabled the identification of shared and unique predictors of traumatic birth and CB-PTSD. In the discussion, we therefore address statistically significant findings but place particular emphasis on those predictors that demonstrate clinical relevance, as reflected in both the magnitude of effect sizes (odds ratios [ORs]/*β*s) and their unique contribution to the variability of the dependent variables.

**Birth experience** was strongly associated with all CB-PTSD outcomes. A more negative experience predicted increased likelihood of CB-PTSD diagnosis, higher symptom severity, and greater perceived traumatic birth, independent of birth mode. Importantly, birth experience also showed the largest and most meaningful part *R*^2^ values across all models, indicating that it accounted for a substantial proportion of unique variance in the outcomes, far exceeding the contribution of other predictors. In addition, its OR for CB-PTSD diagnosis and the beta coefficients for symptom severity and subjective traumatic birth highlight the consistency of this effect across different analytic approaches. This finding underscores that birth experience is not only statistically significant but also the most robust and practically relevant predictor of CB-PTSD in real-world terms. This pattern is consistent with meta-analyses highlighting maternal birth experience as a key predictor of the various aspects of CB-PTSD (Andersen et al., 2012; Ayers et al., 2016; Dekel et al., 2017; El Founti Khsim et al., 2022; Gerkin & O’Hara, 2014; Heyne et al., 2022; Kranenburg et al., 2023).

In addition, mothers who believed that they or their baby might be seriously injured or die during birth (i.e., met criterion A for trauma) had significantly higher CB-PTSD symptoms and traumatic birth perception. This finding highlights the centrality of the perceived threat in CB-PTSD and reflects its gatekeeper role in PTSD diagnostic criteria. Despite ongoing debates on the relevance of criterion A (Marx, Hall-Clark, Friedman, Holtzheimer, & Schnurr, 2024), these results suggest that perceived threat to self and/or infant remains a meaningful contributor to CB-PTSD development. Importantly, the part *R*^2^ values for criterion A were also substantial, indicating that its contribution goes beyond statistical significance. Together with consistent ORs and beta coefficients across outcomes, this shows that criterion A is not only a statistical finding but also a predictor of real-world relevance for maternal mental health. Thus, the perception of danger to the self or the baby plays a significant role in the number and severity of symptoms, as well as in the subjective perception of birth.

We next turn to other predictors that reached statistical significance, albeit with smaller effect sizes. It is important to note that within the current model, these findings represent additional contributions, even if modest, beyond the effects of the other variables.

**Infant complications during birth** and **ongoing maternal complications** were associated with all three outcomes. Mothers reporting serious neonatal complications or persistent own health issues were more likely to report CB-PTSD symptoms and possible diagnosis, and perceive the birth as traumatic. These findings align with previous evidence that concern for infant well-being and unresolved maternal complications intensify trauma responses (Andersen et al., 2012; Ayers et al., 2016; Dekel et al., 2017; Duval et al., 2022; Kranenburg et al., 2023).

Preexisting maternal vulnerabilities were primarily linked to CB-PTSD symptoms and diagnosis. Women with **prior trauma** and **current mental health issues** were more likely to meet diagnostic criteria and report higher symptoms, aligning with the diathesis-stress model, highlighting preexisting variables (Andersen et al., 2012; Ayers et al., 2016; Dekel et al., 2017; El Founti Khsim et al., 2022; Gerkin & O’Hara, 2014; Heyne et al., 2022; Kranenburg et al., 2023). On the other hand, although these vulnerabilities were significantly associated with perceived traumatic birth in the preliminary analysis, in the final model, where birth events and outcomes (such as birth type, birth satisfaction, and health complications) were considered, their association with perceived traumatic birth became insignificant.

Our finding that ongoing maternal or infant complications were associated with CB-PTSD symptoms suggests that physical risk factors may extend into the postpartum period. Need for intensive care or a difficult recovery, such as prolonged maternal healing or neonatal health issues, may intensify childbirth-related stress and hinder psychological recovery, thereby sustaining or worsening CB-PTSD symptoms.

Mode of birth was associated only with perceived traumatic birth: assisted vaginal births and emergency cesareans were linked to higher trauma ratings, and elective cesareans to lower ones. This supports prior findings that vaginal instrumental births or emergency cesarean sections evoke feelings of danger and loss of control (Beck-Hiestermann, Hartung, Richert, Miethe, & Wiegand-Grefe, 2024; Thiel & Dekel., 2020). While earlier studies linked them to CB-PTSD symptoms (Carter, Bick, Gallacher, & Chang, 2022; Ginter et al., 2022), mode of birth did not predict diagnosis or symptoms once the subjective experience of birth was considered. These findings suggest that subjective appraisal may play a greater role in CB-PTSD outcomes than birth mode, although the latter still shapes perceived trauma (Ayers et al., 2016; Andersen et al., 2012). Of note, in our analysis, only emergency cesarean section demonstrated a meaningful effect size, whereas associations for assisted vaginal and elective cesarean births were negligible once effect sizes were taken into account.

Findings on support during birth and the number of children (parity) were partly consistent with previous research. Greater perceived support from companions was associated with lower symptom severity, in line with prior studies (Andersen et al., 2012; Dekel et al., 2017; El Founti Khsim et al., 2022), and multiparity was linked to reduced symptoms, as also reported previously (Ayers et al., 2016; Carter et al., 2022; Chabbert, Panagiotou, & Wendland, 2021). In contrast, the presence of more companions was unexpectedly related to greater symptoms and diagnosis, contradicting earlier findings (Handelzalts, Levy, Ayers, Krissi, & Peled, 2022). However, across all of these variables, the ORs, beta coefficients, and part *R*^2^ values were negligible, suggesting that despite statistical significance, these associations lack clinical relevance.

The finding that multilevel models with random effects for country provided the best fit suggests that associations between risk factors and CB-PTSD outcomes vary across countries – unsurprising given INTERSECT’s international scope. This likely reflects cultural, systemic, and contextual differences in childbirth, healthcare, and social norms. While the identified risk factors reflect overall trends, they may not fully capture country-specific vulnerabilities. Future analyses of the data will examine cross-cultural differences and the contextual relevance of specific predictors.

### Strengths and limitations

This study’s strengths include a large international sample, standardized protocol, and use of a CB-PTSD measure aligned with DSM-5, together with the assessment of probable diagnosis, symptom severity, and perceived trauma, which enabled a comprehensive analysis of risk factors. Limitations include reliance on self-report, with no clinical verification of symptoms or complications; limited inclusion of low-income countries; and samples that were not nationally representative. Survey length also restricted detailed assessment of interventions, complications, and prior trauma or mental health. The cross-sectional design limits causal inference, highlighting the need for future studies with clinical interviews, longitudinal methods, and broader inclusion, particularly from low- and middle-income countries.

## Conclusion

This international study identified key risk factors for CB-PTSD related to diagnosis, symptom severity, and perceived traumatic birth. Despite some variation, negative birth experiences, PTSD criterion A endorsement, and maternal or infant complications consistently indicated a higher risk. These results highlight the importance of **birth factors** within the stress-diathesis framework of childbirth PTSD risk factors. They further highlight the importance of improving birth experiences, as subjective perceptions can be as influential as clinical outcomes. Addressing mothers’ experiences and perceptions of danger to themselves or the baby can be key factors in identifying and possibly preventing the development of childbirth PTSD.

## Supporting information

Handelzalts et al. supplementary material Handelzalts et al. supplementary material

## Data Availability

Data and supporting documentation for the INTERSECT study (2024) are available through the UK Data Service: SN: 9295, DOI: http://doi.org/10.5255/UKDA-SN-9295-1, URL: https://beta.ukdataservice.ac.uk/datacatalogue/studies/study?id=9295.

## Supplementary material

The supplementary material for this article can be found at http://doi.org/10.1017/S0033291725102298.
