# Supplementary material for: Cross-national risk factors for childbirth-related PTSD: Findings from the INTERSECT study
Source: Psychol Med. 2025 Nov 17;55:e349. doi: 10.1017/S0033291725102298 (PMC13058632; doi:10.1017/S0033291725102298)
Supplement: Handelzalts et al. supplementary material [file S0033291725102298sup001.docx]

**Supplementary file:**

eFigure 1. Participant Flow Diagram for Inclusion in the Statistical Analyses

eTable 1- Sample sociodemographic characteristics by country

eTable 2 – Demographic variables fixed effect coefficients for each dependent variable

eTable 3 – Pregnancy variables fixed effect coefficients for each dependent variable.

eTable 4 – Birth variable fixed effect coefficients for each dependent variable.

eTable 5 – Infant variables fixed effect coefficients for each dependent variable.

eTable 6 – SDs of random slopes for each model.

Equation 1 - Model equation for PTSD diagnosis.

Equation 2 - Model equation for symptoms severity (City BiTS total score).

Equation 3 - Model equation for perceived traumatic birth.

**eFigure 1. Participant Flow Diagram for Inclusion in the Statistical Analyses**

Initial sample size (N=11,302)

Participants from Switzerland with missing data on the number of birth companions (N=247)

Data for analysis

(N=11,055)

Analysis of CB-PTSD diagnosis (N=10,116)

Analysis of CB-PTSD symptoms (N=10,130)

Analysis of perceived traumatic birth (N=10,086)

Missing data

(N=939)

Missing data

(N=925)

Missing data

(N=969)

eTable 1- Sample sociodemographic characteristics by country

| **Country** | **N** | **Country’s Income Status ^a^** | **Weeks since birth**  Mean (SD) | **Age**  Mode (%)^b^ | **Education**  Mode (%)^b^ | **Household income**  Mode (%)^b^ | **Relationship status**  Mode (%)^b^ | **Ethnic/racial**  **minority**  % **^c^** | **Not country of birth**  % **^c^** |
| --- | --- | --- | --- | --- | --- | --- | --- | --- | --- |
| Australia | 166 | High | 8.2 (1.7) | 30-34 (33.7) | Higher education (74.5) | Average (69.9) | Married (57.8) | Missing | 38.6 |
| Brazil | 596 | Upper-middle | 8.9 (2.1) | 25-29 (29.5) | High school (66.6) | Average (49.5) | Cohabiting (38.4) | 34.5 | 1.6 |
| Chile | 127 | High | 9.2 (1.7) | 30-34 (36.2) | High school (64.8) | Below average (57.6) | Cohabiting (63.2) | Missing | 32.8 |
| Croatia | 380 | High | 7.7 (1.4) | 30-34 (37.1) | Higher education (73.0) | Average (61.4) | Married (75.8) | 0.3 | 11.1 |
| Cyprus | 142 | High | 7.4 (1.7) | 30-34 (40.8) | Higher education (93.0) | Average (58.2) | Married (90.1) | 8.5 | 23.9 |
| Czechia | 246 | High | 6.9 (1.3) | 25-29 (35.8) 30-34 (35.8) | Higher education (49.8) | Average (77.0) | Married (58.0) | 1.7 | 4.5 |
| Estonia | 285 | High | 8.2 (1.6) | 25-29 (33.7) | Higher education (55.1) | Below average (40.7) | Cohabiting (61.1) | 0.0 | 0.0 |
| Germany | 1644 | High | 10.7 (0.8) | 30-34 (42.9) | Higher education (52.0) | Missing | Married (48.3) | Missing | 8.8 |
| Iceland | 701 | High | 7.5 (1.5) | 30-34 (32.1) | Higher education (72.6) | Average (64.5) | Married (77.1) | 2.5 | 15.0 |
| Ireland | 274 | High | 7.4 (1.5) | 35-39 (37.2) | Higher education (79.6) | Average (66.3) | Married (64.1) | 11.4 | 35.8 |
| Israel | 248 | High | 8.5 (0.5) | 30-34 (39.9) | Higher education (72.6) | Average (34.6) | Married (93.5) | 6.1 | 13.9 |
| Italy | 211 | High | 7.9 (1.8) | 30-34 (42.2) | Higher education (65.0) | Average (77.7) | Married (42.4) | 7.1 | 18.0 |
| Lithuania | 328 | High | 7.1 (1.1) | 30-34 (36.6) | Higher education (75.3) | Average (58.5) | Married (78.4) | 0.6 | 0.9 |
| Malawi | 248 | Least developed | 8.5 (1.6) | 20-24 (25.8) 25-29 (25.8) | Elementary (71.8) | Below average (88.7) | Married (80.2) | Missing | 0.4 |
| Nepal | 490 | Least developed | 7.6 (1.5) | 25-29 (40.8) | High school (38.2) | Below average (48.4) | Married (93.5) | 28.8 | 10.6 |
| Nigeria | 406 | Lower-middle | 7.8 (1.9) | 25-29 (31.5) | High school (58.0) | Average (44.0) | Married (67.4) | 8.6 | 2.2 |
| Norway | 221 | High | 8.4 (1.8) | 30-34 (45.2) | Higher education (91.0) | Average (59.7) | Cohabiting (56.1) | 3.2 | 6.8 |
| Pakistan | 335 | Lower-middle | 9.2 (1.1) | 25-29 (40.9) | Higher education (37.3) | Average (58.5) | Married (100.0) | 0.6 | 0 |
| Poland | 296 | High | 10.9 (0.8) | 30-34 (36.5) | Higher education (73.0) | Average (64.2) | Married (70.3) | 2.4 | 2.4 |
| Portugal | 227 | High | 7.7 (1.7) | 35-39 (32.2) | Higher education (65.9) | Average (70.6) | Married (76.4) | 1.9 | 15.5 |
| Romania | 135 | High | 7.5 (1.7) | 30-34 (32.6) | Higher education (78.5) | Average (60.7) | Married (87.4) | 3.0 | 6.7 |
| Saudi Arabia | 248 | High | 10.2 (1.9) | 30-34 (31.9) | Higher education (67.5) | Average (70.5) | Married (99.2) | 5.1 | 5.9 |
| Serbia | 267 | Upper-middle | 8.8 (1.8) | 30-34 (38.6) | Higher education (68.9) | Average (65.9) | Married (93.6) | 0.7 | 8.6 |
| Slovakia | 437 | High | 7.5 (1.0) | 30-34 (38.9) | Higher education (58.2) | Average (83.2) | Married (65.4) | 0.7 | 1.8 |
| Slovenia | 236 | High | 8.2 (1.9) | 40+ (30.5) | Higher education (78.7) | Average (67.2) | Cohabiting (55.2) | 2.3 | 3.4 |
| Spain | 254 | High | 8.9 (1.5) | 30-34 (38.2) | Higher education (72.4) | Average (72.4) | Married (61.4) | 7.5 | 9.1 |
| Sweden | 469 | High | 9.4 (1.5) | 30-34 (45.0) | Higher education (80.0) | Average (54.3) | Cohabiting (57.8) | 9.2 | 12.0 |
| Switzerland | 247 | High | 6.5 (1.1) | 30-34 (53.0) | Higher education (70.9) | Average (68.8) | Married (59.5) | 5.3 | 37.2 |
| Türkiye | 1013 | Upper-middle | 7.6 (1.3) | 25-29 (45.5) | Higher education (47.3) | Average (65.6) | Married (97.3) | 19.7 | 8.7 |
| UAE | 165 | High | 7.6 (1.6) | 30-34 (38.2) | Higher education (87.9) | Average (57.6) | Married (100.0) | 14.5 | 27.3 |
| UK | 260 | High | 8.2 (1.7) | 30-34 (30.8) | Higher education (73.8) | Average (56.4) | Married (57.8) | 17.6 | 24.9 |
| Total sample |  |  | 8.5 (1.9) | 30-34 (33.6) | Higher education (57.1) | Average (58.5) | Married (67.6) | 9.7 | 10.4 |
| Total N |  |  | 11302 | 11302 | 11058 | 10938 | 11081 | 8858 | 11066 |

^a^ Countries’ income status according to the Official Development Assistance (ODA) category used by the Development Assistance Committee (DAC) of the Organisation for Economic Co-operation and Development; ^b^ mode for each country and the percentage of the sample that were in that modal group; ^c^ percentage of each sample who were ethnic minorities or not living in their country of birth; UAE, United Arab Emirates; UK, United Kingdom·

**Variables selection process:**

**Demographic variables:**

All models included random intercept and slopes with diagonal structure. For CB-PTSD diagnosis, model comparison revealed that a model with random intercept and slopes in diagonal covariance structure (AIC = 4147.7, BIC = 4378.8) had better fit than a model with a random intercept only (AIC = 4183.0, BIC = 4334.7, χ^2^(11) = 57.33, p < .001). The unrestricted model did not present better fit (AIC = 4435.7, BIC = 6096.7, χ^2^(198) = 108.00, p = 1.000). As for CB-PTSD symptoms, model comparison revealed that a model with random intercept and slopes in diagonal covariance structure (AIC = 74560, BIC = 74820) had better fit than a model with a random intercept only (AIC = 74833, BIC = 74992, χ^2^(14) = 301.41, p < .001). The unrestricted model did not present better fit (AIC = 74749, BIC = 76417, χ^2^(195) = 200.91, p = .370). Finally, for perceived traumatic birth, model comparison revealed that a model with random intercept and slopes in diagonal covariance structure (AIC = 49832, BIC = 50078) had better fit than a model with a random intercept only (AIC = 49943, BIC = 50102, χ^2^(12) = 134.73, p < .001). The unrestricted model did not present better fit (AIC = 50107, BIC = 51775, χ^2^(197) = 119.38, p = 1.000).

eTable 2 – Demographic variables fixed effect coefficients for each dependent variable.

|  | **CB-PTSD diagnosis** | | | | **CB-PTSD symptoms** | | | | **Perceived traumatic birth** | | | |
| --- | --- | --- | --- | --- | --- | --- | --- | --- | --- | --- | --- | --- |
|  | **B** | **95% CI** | **z** | **p** | **B** | **95% CI** | **t** | **p** | **B** | **95% CI** | **t** | **p** |
| Intercept | -2.62 | -3.22, -2.02 | -8.53 | <.001 | 17.12 | 14.93, 19.31 | 15.30 | <.001 | 4.27 | 3.71, 4.83 | 14.92 | <.001 |
| Age | -0.01 | -0.12, 0.10 | -0.12 | .903 | -0.28 | -0.67, 0.12 | -1.38 | .169 | 0.02 | -0.06, 0.10 | 0.52 | .603 |
| Education | -0.11 | -0.29, 0.06 | -1.29 | .197 | 0.22 | -0.19, 0.63 | 1.05 | .294 | 0.02 | -0.15, 0.20 | 0.23 | .821 |
| Income | 0.06 | -0.14, 0.27 | 0.61 | .540 | -0.25 | -0.73, 0.24 | -1.00 | .316 | -0.05 | -0.14, 0.05 | -0.99 | .321 |
| Number of children | -0.21 | -0.35, -0.07 | -2.97 | .003 | -0.90 | -1.31, -0.49 | -4.29 | <.001 | 0.56 | -0.69, -0.42 | -8.09 | <.001 |
| Immigrant | -0.08 | -0.31, 0.15 | -0.68 | .499 | 0.21 | -0.40, 0.82 | 0.67 | .503 | 0.07 | -0.09, 0.23 | 0.85 | .398 |
| Resident area |  |  |  |  |  |  |  |  |  |  |  |  |
| City | 0.06 | -0.12, 0.24 | 0.68 | .496 | 0.38 | -0.13, 0.88 | 1.47 | .142 | -0.01 | -0.11, 0.09 | -0.23 | .821 |
| Town (ref.) | - | - | - | - | - | - | - | - | - | - | - | - |
| Rural area | -0.04 | -0.26, 0.19 | -0.33 | .743 | -0.22 | -0.65, 0.21 | -0.99 | .322 | -0.01 | -0.12, 0.14 | -0.17 | .868 |
| Relationship status |  |  |  |  |  |  |  |  |  |  |  |  |
| Married (ref.) | - | - | - | - | - | - | - | - | - | - | - | - |
| Partner | -0.09 | -0.48, 0.29 | -0.48 | .629 | -1.09 | -2.15, -0.04 | -2.03 | .042 | -0.17 | -0.49, 0.14 | -1.08 | .278 |
| In relationship | -0.40 | -1.08, 0.29 | -1.14 | .256 | 0.07 | -1.62, 1.75 | 0.08 | .939 | 0.27 | -0.23, 0.76 | 1.06 | .289 |
| Single | -0.07 | -0.57, 0.42 | -0.29 | .769 | -1.16 | -2.52, 0.21 | -1.66 | .098 | -0.07 | -0.50, 0.35 | -0.35 | .730 |
| Widowed | 0.36 | -0.98, 1.69 | 0.52 | .603 | 2.96 | -1.38, 7.30 | 1.34 | .182 | -0.02 | -1.31, 1.28 | -0.02 | .982 |
| Separated / divorced | 0.36 | -0.38, 1.10 | 0.95 | .342 | 0.38 | -1.61, 2.36 | 0.37 | .711 | 0.17 | -0.43, 0.77 | 0.56 | .576 |
| Any previous trauma | 0.43 | 0.28, 0.58 | 5.53 | <.001 | 1.55 | 1.14, 1.96 | 7.45 | <.001 | 0.27 | 0.17, 0.36 | 5.49 | <.001 |
| Previous mental health diagnosis | 0.23 | 0.06, 0.39 | 2.68 | .007 | 0.27 | -0.26, 0.80 | 1.01 | .315 | 0.10 | -0.04, 0.23 | 1.42 | .156 |
| Current mental health diagnosis | 0.50 | 0.33, 0.67 | 5.86 | <.001 | 3.46 | 2.84, 4.07 | 10.98 | <.001 | 0.30 | 0.10, 0.49 | 3.03 | .002 |
| Currently receiving mental health treatment | -0.06 | -0.40, 0.27 | -0.37 | .711 | 1.30 | 0.39, 2.21 | 2.81 | .005 | -0.01 | -0.26, 0.25 | -0.05 | .961 |
| Previously received mental health treatment | 0.23 | 0.01, 0.45 | 2.07 | .038 | 0.93 | 0.37, 1.48 | 3.29 | .001 | 0.07 | -0.12, 0.26 | 0.69 | .492 |
| Type of mental health treatment |  |  |  |  |  |  |  |  |  |  |  |  |
| Psychotherapy (ref.) | - | - | - | - | - | - | - | - | - | - | - | - |
| Medication | 0.12 | -0.36, 0.60 | 0.48 | .633 | -0.33 | -1.59, 0.94 | -0.51 | .614 | -0.02 | -0.29, 0.24 | -0.17 | .867 |
| Both | -0.21 | -0.71, 0.29 | -0.82 | .414 | 0.91 | -0.27, 2.09 | 1.52 | .129 | 0.07 | -0.22, 0.37 | 0.50 | .621 |

**Pregnancy variables:**

For CB-PTSD diagnosis, model comparison revealed that a model with random intercept and slopes in diagonal covariance structure (AIC = 4364.6, BIC = 4408.0) had better fit than a model with a random intercept only (AIC = 4374.6, BIC = 4403.5, χ^2^(2) = 13.96, p < .001). A model with random intercept and slopes in unrestricted covariance structure did not have a better fit (AIC = 4364.7, BIC = 4429.6, χ^2^(3) = 5.98, p = .112). As for CB-PTSD symptoms, model comparison revealed that a model with random intercept and slopes in unrestricted covariance structure (AIC = 75667, BIC = 75739) had better fit than both a model with a random intercept only (AIC = 75852, BIC = 75888, χ^2^(5) = 195.29, p < .001), and a model with random intercept and slopes in diagonal covariance structure (AIC = 75684, BIC = 75735, χ^2^(3) = 23.46, p < .001). Finally, for perceived traumatic birth, model comparison revealed that a model with random intercept and slopes in diagonal covariance structure (AIC = 50294, BIC = 50344) had better fit than a model with a random intercept only (AIC = 50304, BIC = 50340, χ^2^(2) = 13.86, p < .001). The unrestricted model did not present better fit (AIC = 50295, BIC = 50368, χ^2^(3) = 4.29, p = .232).

eTable 3 – Pregnancy variables fixed effect coefficients for each dependent variable.

|  | **CB-PTSD diagnosis** | | | | **CB-PTSD symptoms** | | | | **Perceived traumatic birth** | | | |
| --- | --- | --- | --- | --- | --- | --- | --- | --- | --- | --- | --- | --- |
|  | **B** | **95% CI** | **z** | **P** | **B** | **95% CI** | **T** | **p** | **B** | **95% CI** | **t** | **p** |
| Intercept | -2.88 | -3.24, -2.51 | -15.55 | <.001 | 11.19 | 9.46, 12.92 | 12.69 | <.001 | 3.54 | 3.07, 4.01 | 14.84 | <.001 |
| Previous pregnancy loss | 0.13 | -0.01, 0.28 | 1.78 | .075 | 0.30 | -0.18, 0.78 | 1.24 | .216 | -0.03 | -0.12, 0.06 | -0.63 | .528 |
| Previous birth trauma | 0.18 | 0.05, 0.31 | 2.78 | .005 | 1.17 | 0.56, 1.78 | 3.78 | <.001 | -0.04 | -0.16, 0.07 | -0.76 | .446 |

**Birth variables:**

For CB-PTSD diagnosis, model comparison revealed that a model with random intercept and slopes in diagonal covariance structure (AIC =2242.7, BIC = 2365.5) had better fit than a model with a random intercept only (AIC = 2312.3, BIC = 2406.2, χ^2^(4) = 77.54, p < .001). A model with random intercept and slopes in unstructured covariance structure did not have better performance (AIC = 2391.7, BIC = 2875.5, χ^2^(50) = 0.00, p = 1.000). As for CB-PTSD symptoms, model comparison revealed that a model with random intercept and slopes in diagonal covariance structure (AIC = 70575, BIC = 70749) had better fit than a model with a random intercept only (AIC = 71694, BIC = 71795, χ^2^(10) = 1138.73, p < .001). A model with random intercept and slopes with unstructured covariance structure was singular and therefore was not compared. Finally, for perceived traumatic birth, model comparison revealed that a model with random intercept and slopes in diagonal covariance structure (AIC = 44043, BIC = 44217) had better fit than both a model with a random intercept only (AIC = 44634, BIC = 44735, χ^2^(10) = 610.53, p < .001), and an unrestricted model (AIC = 44072, BIC = 44729, χ^2^(67) = 105.31, p = .002).

eTable 4 – Birth variable fixed effect coefficients for each dependent variable.

|  | **CB-PTSD diagnosis** | | | | **CB-PTSD symptoms** | | | | **Perceived traumatic birth** | | | |
| --- | --- | --- | --- | --- | --- | --- | --- | --- | --- | --- | --- | --- |
|  | **B** | **95% CI** | **z** | **p** | **B** | **95% CI** | **t** | **p** | **B** | **95% CI** | **t** | **p** |
| Intercept | -4.22 | -4.78, -3.65 | -14.57 | <.001 | 14.33 | 12.61, 16.04 | 16.38 | <.001 | 4.48 | 4.10, 4.85 | 23.34 | <.001 |
| Birth method |  |  |  |  |  |  |  |  |  |  |  |  |
| Vaginal (ref.) | - | - | - | - | - | - | - | - | - | - | - | - |
| Assisted vaginal | 0.12 | -0.20, 0.44 | 0.76 | .446 | 0.24 | -0.33, 0.80 | 0.82 | .413 | 0.35 | 0.20, 0.50 | 4.59 | <.001 |
| Emergency caesarean | 0.03 | -0.18, 0.24 | 0.28 | .783 | 0.12 | -0.36, 0.60 | 0.49 | .626 | 0.38 | 0.22, 0.55 | 4.65 | <.001 |
| Elective caesarean | -0.17 | -0.46, 0.12 | -1.14 | .253 | -0.39 | -0.77, -0.01 | -2.00 | .046 | -0.64 | -0.79, -0.48 | -8.22 | <.001 |
| Maternal complications during birth |  |  |  |  |  |  |  |  |  |  |  |  |
| No (ref.) | - | - | - | - | - | - | - | - | - | - | - | - |
| Major | -0.01 | -0.25, 0.23 | -0.05 | .959 | 0.53 | 0.00, 1.05 | 1.97 | .049 | 0.41 | 0.26, 0.56 | 5.39 | <.001 |
| Minor | 0.06 | -0.11, 0.23 | 0.68 | .495 | -0.00 | -0.29, 0.29 | -0.03 | .979 | -0.07 | -0.15, 0.00 | -1.93 | .053 |
| Ongoing maternal complications | 0.28 | 0.12, 0.44 | 3.49 | <.001 | 1.96 | 1.40, 2.53 | 6.83 | <.001 | 0.10 | 0.00, 0.20 | 2.04 | .041 |
| During birth – believed her or the baby injured | - | - | - | - | 1.54 | 1.05, 2.03 | 6.14 | <.001 | 0.36 | 0.22, 0.50 | 5.13 | <.001 |
| During birth – believed her or the baby would die | - | - | - | - | 2.03 | 1.33, 2.73 | 5.69 | <.001 | 0.45 | ,0.32, 0.57 | 6.98 | <.001 |
| Number of birth companions | 0.33 | 0.06, 0.59 | 2.43 | .015 | 1.92 | 1.09, 2.75 | 4.55 | <.001 | -0.09 | -0.27, 0.10 | -0.90 | .367 |
| Level of support from birth companion | -0.21 | -0.36, -0.07 | -2.86 | .004 | -0.60 | -0.94, -0.26 | -3.43 | <.001 | 0.04 | -0.04, 0.12 | 0.92 | .356 |
| Birth experience | -0.16 | -0.18, -0.13 | -11.97 | <.001 | -0.51 | -0.59, -0.42 | -11.39 | <.001 | -0.21 | -0.24, -0.18 | -14.60 | <.001 |

**Infant variables:**

For CB-PTSD diagnosis, model comparison revealed that both a model with random intercept and slopes in unstructured covariance structure (AIC = 4158.6, BIC = 4303.1) and a model with random intercept and slopes in diagonal covariance structure (AIC = 4152.4, BIC = 4217.4), did not have significantly better fit than a model with a random intercept only (AIC = 4153.5, BIC = 4196.8; χ^2^(14) = 22.82, p = .063; χ^2^(3) = 7.10, p = .069, respectively). As for CB-PTSD symptoms, model comparison revealed that a model with random intercept and slopes in unstructured covariance structure (AIC = 75443, BIC = 75595) had better fit than both a model with a random intercept only (AIC = 75508, BIC = 75559, χ^2^(14) = 93.06, p < .001), and a model with random intercept and slopes with diagonal covariance structure (AIC = 75470, BIC = 75550, χ^2^(10) = 47.20, p < .001). Finally, for perceived traumatic birth, model comparison revealed that a model with random intercept and slopes in diagonal covariance structure (AIC = 49795, BIC = 49875) had better fit than a model with a random intercept only (AIC = 49881, BIC = 49931, χ^2^(4) = 93.36, p < .001). The unrestricted model did not present a better fit (AIC = 49805, BIC = 49957, χ^2^(10) = 10.37, p = .409).

eTable 5 – Infant variables fixed effect coefficients for each dependent variable.

|  | **CB-PTSD diagnosis** | | | | **CB-PTSD symptoms** | | | | **Perceived traumatic birth** | | | |
| --- | --- | --- | --- | --- | --- | --- | --- | --- | --- | --- | --- | --- |
|  | **B** | **95% CI** | **z** | **p** | **B** | **95% CI** | **t** | **p** | **B** | **95% CI** | **t** | **p** |
| Intercept | -2.15 | -2.53, -1.78 | -11.25 | <.001 | 14.90 | 12.91, 16.89 | 14.69 | <.001 | 4.82 | 4.37, 5.26 | 21.09 | <.001 |
| Infant complications during birth |  |  |  |  |  |  |  |  |  |  |  |  |
| No (ref.) | - | - | - | - | - | - | - | - | - | - | - | - |
| Major | 0.76 | 0.56, 0.97 | 7.38 | <.001 | 3.21 | 2.09, 4.33 | 5.61 | <.001 | 1.06 | 0.81, 1.23 | 8.45 | <.001 |
| Minor | 0.06 | -0.09, 0.21 | 0.78 | .436 | -0.07 | -0.79, 0.65 | -0.20 | .0844 | 0.02 | -0.14, 0.17 | 0.21 | .838 |
| Ongoing Infant complications | 0.42 | 0.26, 0.57 | 5.31 | <.001 | 2.40 | 1.44, 3.64 | 4.88 | <.001 | 0.44 | 0.22, 0.66 | 3.89 | <.001 |
| Gestation | -0.02 | -0.06, 0.02 | -1.17 | .243 | -0.08 | -0.25, 0.09 | -0.88 | .379 | 0.04 | -0.03, 0.10 | 1.11 | .266 |

eTable 6 – SDs of random slopes for each model.

|  | CB-PTSD diagnosis | CB-PTSD symptoms | Perceived traumatic birth |
| --- | --- | --- | --- |
| Number of children | 0.01 | 0.50 |  |
| Any previous trauma | 0.29 | 0.36 | 0.12 |
| Current mental health diagnosis | 0.27 | 0.72 | 0.16 |
| Previous traumatic birth |  | 0.70 |  |
| Birth method – emergency caesarean |  |  | 0.32 |
| Birth method - elective caesarean |  |  | 0.25 |
| Major maternal complications during birth |  | 0.77 | 0.18 |
| Ongoing maternal complications | 0.09 | 1.13 |  |
| During birth – believed she or the baby injured |  | 0.96 | 0.32 |
| During birth – believed she or the baby would die |  | 1.58 | 0.25 |
| Number of birth companions |  | 1.24 |  |
| Level of support from birth companion | 0.25 | 0.76 |  |
| Birth experience | 0.04 | 0.21 | 0.07 |
| Major infant complications during birth | 0.32 |  |  |
| Ongoing Infant complications | 0.23 | 1.26 |  |

**Equation 1 - Model equation for PTSD diagnosis**

*~ Number of children+ any previous trauma+ previous mental health diagnosis+ current mental health diagnosis+previously received mental health treatment+previous birth trauma+ongoing maternal complications+ number of birth companions+level of support from birth companion+ birth experince+major infant complications during the birth+ ongoing infant complications +(number of children | country)+ (any previous trauma | country) + (current mental health diagnosis | country)+ (level of support from birth companion | country)+(ongoing maternal complications | country)+ (birth experience | country)+(major infant complications during the birth | country)+(ongoing infant complications | country)*

**Equation 2 - Model equation for symptoms severity (City BiTS total score)**

*~number of children+relationship status partner+any previous trauma+current mental health diagnosis+currently received mental health treatment+previously received mental health treatment+previous birth trauma+birth method elective caesarean+major maternal complications during birth+ ongoing maternal complications+during birth believed her or the baby injured+during birth believed her or the baby would die+number of birth companions+level of support from birth companion+birth experince+major infant complications+ongoing infant complications +(number of children | country)+(any previous trauma | country)+(current mental health diagnosis | country)+(previous birth trauma | country)+(major maternal complications during birth | country)+(ongoing maternal complications | country)+(during birth believed her or the baby injured | country)+(during birth believed her or the baby would die | country)+(number of birth companions | country)+(level of support from birth companion | country)+(birth experience| country)+(ongoing infant complications | country)*

**Equation 3 - Model equation for perceived traumatic birth**
*~number of children+any previous trauma+current mental health diagnosis+birth method assisted vaginal+birth method emergency caesarean+birth method elective caesarean+major mother complications during the birth+ongoing maternal complications+during birth believed her or the baby injured+during birth believed her or the baby would die+birth experince+major infant complications during the birth+ongoing infant complications+(any previous trauma | country)+(current mental health diagnosis | country)+(birth method emergency caesarean | country)+(birth method elective caesarean | country)+(major mother complications during the birth | country)+(during birth believed her or the baby injured | country)+(during birth believed her or the baby would die | country)+(birth experience | country)*
